# Predictors of serious infections in rheumatoid arthritis—a prospective Brazilian cohort

**DOI:** 10.1186/s42358-024-00363-1

**Published:** 2024-03-29

**Authors:** Ana Luisa Bagno de Almeida, Maria Fernanda B. Resende Guimarães, Maria Raquel da Costa Pinto, Leticia Rocha Pereira, Ana Paula Monteiro Gomides Reis, Karina Rossi Bonfiglioli, Paulo Louzada-Junior, Rina Dalva Neubarth Giorgi, Gláucio Ricardo Werner de Castro, Sebastião Cezar Radominski, Claiton Viegas Brenol, Alisson Pugliesi, Licia Maria Henrique da Mota, Geraldo da Rocha Castelar-Pinheiro

**Affiliations:** 1 Departamento de Reumatologia, Universidade Federal de Minas Gerais, Belo Horizonte, Brazil; 2 Departamento de Reumatologia, Universidade do Estado do Rio de Janeiro, Rio de Janeiro, Brazil; 3 Departamento de Reumatologia, Universidade de Brasília, Brasília, Brazil; 4 Departamento de Reumatologia, Faculdade de Medicina, Universidade de São Paulo, São Paulo, SP Brazil; 5 Departamento de Reumatologia, Universidade de São Paulo, Ribeirão Preto, Brazil; 6 Departamento de Reumatologia, Hospital do Servidor Público Estadual de São Paulo, São Paulo, Brazil; 7 Departamento de Reumatologia, Universidade do Sul de Santa Catarina-Unisul, Florianópolis, Brazil; 8 Departamento de Reumatologia, Universidade Federal do Paraná, Curitiba, Brazil; 9 Departamento de Reumatologia, Universidade Federal do Rio Grande do Sul, Porto Alegre, Brazil; 10 Departamento de Reumatologia, Universidade Estadual de Campinas, Campinas, SP Brazil

**Keywords:** Rheumatoid arthritis, Serious infection, Corticosteroids, Brazilian cohort

## Abstract

**Background:**

Infections increase mortality and morbidity and often limit immunosuppressive treatment in rheumatoid arthritis patients.

**Objective:**

To analyze the occurrence of serious infections and the associated factors in a cohort of rheumatoid arthritis patients under real-life conditions.

**Methods:**

We analyzed data from the REAL, a prospective observational study, that evaluated Brazilian RA patients, with clinical and laboratory data collected over a year. Univariate and multivariate analyses were performed from the adjustment of the logistic regression model *Generalized Estimating Equations* (GEE), with the primary outcome being the occurrence of serious infection, defined as need for hospitalization or use of intravenous antibiotics for its treatment.

**Results:**

841 patients were included with an average follow-up time of 11.2 months (SD 2.4). Eighty-nine serious infections occurred, corresponding to 13 infections per 100 patient-years. Pulmonary fibrosis, chronic kidney disease (CKD) and central nervous system disease increased the chances of serious infection by 3.2 times (95% CI: 1.5–6.9), 3.6 times (95% CI: 1.2–10.4) and 2.4 times (95% CI: 1.2–5.0), respectively. The use of corticosteroids in moderate doses increased the chances by 5.4 times (95% CI: 2.3–12.4), and for each increase of 1 unit in the health assessment questionnaire (HAQ), the chance increased 60% (95% CI: 20–120%).

**Conclusion:**

The use of corticosteroids at moderate doses increased the risk of serious infection in RA patients. Reduced functionality assessed by the HAQ and comorbidities were other important factors associated with serious infection in this cohort.

**Supplementary Information:**

The online version contains supplementary material available at 10.1186/s42358-024-00363-1.

## Introduction

Rheumatoid arthritis (RA) is a chronic inflammatory disease with an estimated global prevalence of 0.5–1%. In Brazil, this prevalence is close to the lower limit of this range [1, 2].

Studies carried out in different countries have shown an increased risk of infection among patients with RA. The estimated risk is twice as high in this population compared to subjects without RA, and the risk of serious infections (SI) requiring hospitalization is also higher [3, 4]. Infections increase mortality and morbidity and often limit immunosuppressive treatment [5–7]. The risk of infection in this population may be influenced by treatment, the characteristics of rheumatic disease and the clinical and social conditions of the patients [8–14].

Prospective national data in real-life studies assessing infections in patients with RA are still scarce. The most recent studies are limited to evaluate the risk associated with the use of biologic and small molecule drugs [15, 16]. The REAL cohort itself, a multicentric prospective study from which these data was drawn, has already been analyzed regarding the sociodemographic [17, 18] profile, cardiovascular comorbidities [19], treatment [20, 21] and disease activity [22] in RA patients, but this analysis has not included infections events.

Given the large therapeutic arsenal currently available for the treatment of RA, we believe that it is extremely important to understand the risk factors associated with serious adverse effects to safely choose the ideal treatment for each patient. Thus, this study aims to evaluate the incidence and factors related to the occurrence of SI, defined as the need for hospitalization or use of intravenous antibiotics for its treatment, among patients with RA under real-life conditions in Brazil.

## Methods

### Study design and setting

We analyzed data from the national multicenter prospective observational cohort, the REAL study [18], collected between August 2015 and May 2017. Eleven tertiary health care centers in Brazil based in four macroregions of the country participated in this cohort. Each center was expected to enroll 100 patients consecutively. All participating centers are part of the Unified Health System (*Sistema Único de Saúde* or *SUS*, in Portuguese), the Brazilian public health system, and have specialized rheumatology care. Three visits were conducted: the inclusion to the study, the second one (between five and seven months after the first visit), and the third one (between eleven and thirteen months after the inclusion visit).

### Participants

**Fig. 1:**
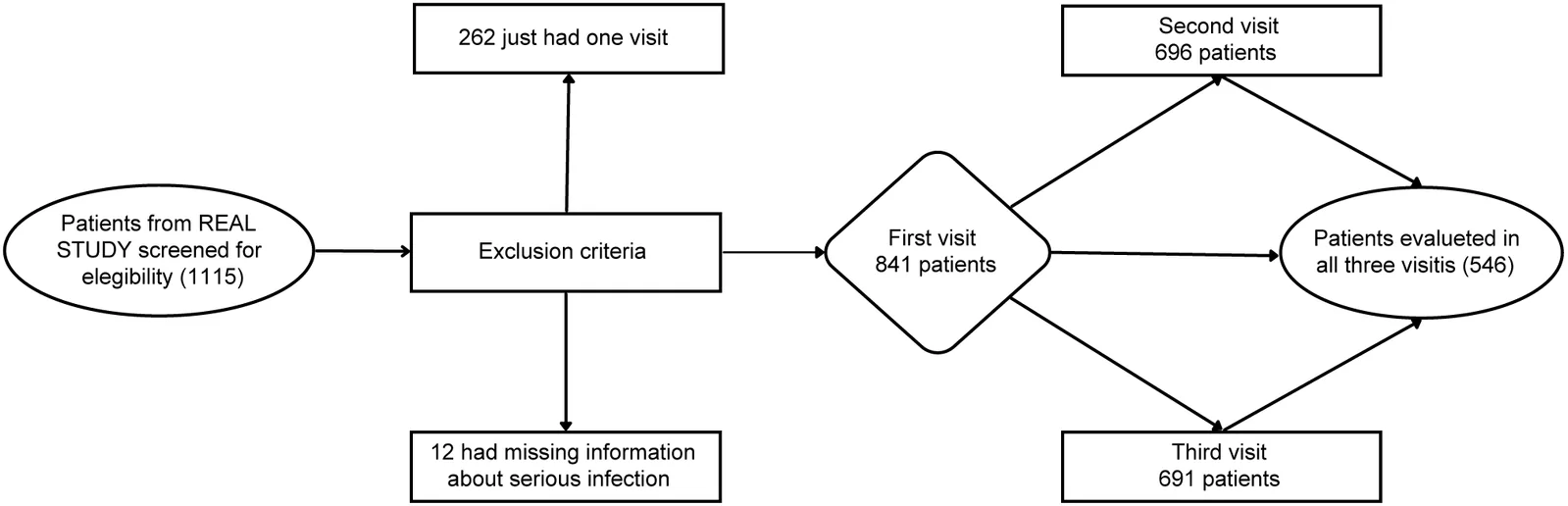
Flowchart

All participants were older than 18 years and met American College of Rheumatology (ACR) 1987 or American College of Rheumatology and European League Against Rheumatism (ACR/EULAR) 2010 classification criteria for RA and were previously followed up in rheumatology services for at least 6 months before inclusion. Initially, information from 1115 patients were collected. However, 274 patients were excluded due to lack of information regarding the infectious events in two or more visits during follow-up. Thus, 841 patients were included with an average follow-up time of 11.2 months (SD 2.4). All patients attended the first visit. After that, 150 patients attended only the 6-months visit and 145 patients attended only the 12-month visit, resulting in 696 and 691 patients at each visit, respectively (Fig. 1).

This study was approved by the local Research Ethics Committee (Comissão de Ética em Pesquisa—COEP)—Federal University of Minas Gerais. All the REAL study participating centers received approval from their Institutions’ Ethics Committees. Signed informed consent was obtained from every study participant.

### Variables and data sources

All data were extracted from the database prepared and provided by the coordination of the REAL study. In da Rocha Castelar-Pinheiro et al. [18] it is possible to see the structure of the cohort and details about the data collected.

For statistical analysis, the following data were extracted: sex, age, socioeconomic status, lifestyle habits including smoking and alcoholism, presence of extra-articular manifestations (EAMs), presence of comorbidities, Charlson comorbidity index, history of previous infections, use of medications, time of disease duration, disease activity indices measured by the Disease Activity Score 28 ESR and CRP (DAS 28 ESR, DAS 28 CRP) and Clinical Disease Activity Index (CDAI), functionality index measured by the Health Assessment Questionnaire (HAQ), number of swollen and tender joints, serologic—rheumatoid factor and anti-cyclic citrullinated peptide antibody (anti-CCP)—and inflammatory tests—C-reactive protein (CRP) and erythrocyte sedimentation rate (ESR).

Comorbidities were grouped into categories and patients could be included in any of them if they had at least one of the diagnoses. These groups were: cardiovascular disease, pulmonary disease, metabolic disease, chronic kidney disease, gastrointestinal tract disease, central nervous system disease, neoplastic diseases, osteoporosis, acquired immunodeficiency syndrome (AIDS) and obesity. Due to its clinical significance, the presence of pulmonary fibrosis as EAM was evaluated in the “extra-articular manifestation” group and as a separate variable.

The variables age, socioeconomic level, dose of corticosteroids and disease activity indices—DAS28 and CDAI—were evaluated in categories and as continuous variables. Disease duration, Charlson comorbidity index and Charlson comorbidity index adjusted for age, CRP and ESR levels, and HAQ were evaluated as continuous variables.

Serious infections were defined as the need for hospitalization or the use of intravenous antibiotics for the treatment of the infection. Information about infections were self-reported and based on medical reports.

### Statistical methods

The results of the descriptive analysis were obtained using measures of central tendency (mean) and measures of dispersion (standard deviation) for quantitative variables. For qualitative variables, frequencies and percentages were obtained. These results were presented by visit for variables with potential for variation over time.

For univariate analysis, comparisons over time (defined as first, second and third visit) were performed from the adjustment of the logistic regression model *Generalized Estimating Equations* (GEE) [23]. Patients with missing information of any variable were excluded from the adjustments of the univariate models.

Variables with p values lower than 0.20 in the univariate analysis were indicated to compose the multivariate model of the initial regression unless they were collinear with another variable. In the case of collinearity, the most statistically significant and clinically appropriate variable was included in the multivariable analysis. Only characteristics with a p value equal to or less than 0.05 remained in the final adjustment. The associations found were quantified from the *odds ratio* (OR) of the model and its respective 95% confidence interval.

The groups of comorbidities with a significant *p* value (*p* < 0.20) were submitted to a further univariate analysis that comprised comorbidities of each group separately to assess the relation to SI. GEE logistic regression model adjustments were performed. We assessed the corticosteroid dose that was most associated with the occurrence of SI maximizing the sum of sensitivity and specificity for each equivalent dose of prednisolone.

Analyses were performed in R version 3.6.3, PASW Statistics 18, and MINITAB 17 software.

## Results

Among the 841 patients eligible for the study, 757 (90.0%) were female, and 324 (38.5%) were 60 years of age or older. The mean age at the beginning of the study was 56 years (SD 11.4 years). On average, patients had been diagnosed with RA for 14 years (SD 9.5 years), showing the greater number of patients with established RA and a long disease duration in this cohort. Considering EAMs, 151 (18%) patients had at least one extra-articular manifestation, and 34 (4%) had pulmonary fibrosis. The sociodemographic characteristics of the study population and the presence of comorbidities and EAMs can be found in Table 1. Regarding treatment, on average of the three visits, 47.6% of patients used corticosteroids, and 92.6% used doses smaller than the equivalent of 10 mg of prednisolone per day. Approximately 90% of patients were using synthetic disease-modifying antirheumatic drugs (DMARDs) and half of them (56.7% on average of the three visits) were using it in monotherapy. Approximately 40% of patients were using biological or synthetic target specific DMARDs (Table 2). Most patients had moderate disease activity, and the mean HAQ over the period was 0.851 (Table 3).

**Table 1:** Baseline clinical and sociodemographic patient characteristics*

Variables	N (%) n = (841)
Female gender	757 (90%)
Age ≥ 60 years	324 (38.5%)
Smoking: ex-user or user	323 (38.4%)
Alcoholism: ex-user or user	120 (14.3%)
Presence of extra-articular manifestations	151 (18%)
Extra-articular manifestations: Pulmonary fibrosis	34 (4%)
Cardiovascular disease	454 (54%)
Pulmonary disease	72 (8.6%)
Gastrointestinal tract disease	52 (6.2%)
Chronic kidney disease	17 (2%)
Metabolic diseases	128 (15.2%)
Osteoporosis	227 (27%)
Central nervous system diseases	68 (8.1%)
Neoplasms	19 (2.3%)
AIDS	0 (0%)
History of previous infection	16 (1.9%)
Obesity	266 (32.3%)^1^
Seropositive disease	687 (82.7%)^2^

*Variables present in this table were collected only in the first visit (baseline)

^1^Missing information = 17

^2^Missing information = 10

*N* number of observations, *%* percentage, *AIDS* acquired immunodeficiency syndrome

**Table 2:** Description of drugs in use for rheumatoid arthritis treatment per visit

Drug in use	Visit 1 N (%) (n = 841)	Visit 2 N (%) (n = 696)	Visit 3 N (%) (n = 691)
Corticosteroid	390 (46.4%)	293 (44%)	361 (52.4%)
Missing information	0	30	2
Oral corticosteroid dose* < 10 mg/day	779 (92.6%)	629 (94.4%)	627 (91%)
≥ 10 mg/day	62 (7.4%)	37 (5.6%)	62 (9%)
≥ 15 mg/day	15 (1.8%)	9 (1.4%)	18 (2.6%)
Synthetic DMARD	764 (90.8%)	620 (90.1%)	620 (90.5%)
Missing information	0	8	6
Exclusive synthetic DMARD	498 (59.2%)	393 (56.5%)	376 (54.4%)
Biological DMARD and small molecules	317 (37.7%)	275 (42.1%)	297 (44.7%)
Missing information	0	42	27
Anti-TNF	165 (19.6%)	147 (22.5%)	154 (23.2%)
Missing information	0	42	27
Exclusive biologicals and small molecules	51 (6.1%)	48 (6.9%)	53 (7.7%)
Synthetic DMARD in combination with biologicals or small molecules	266 (31.6%)	227 (32.6%)	244 (35.3%)

*Prednisolone equivalent dose

*N* number of observations, *%* percentage, *DMARD* disease-modifying antirheumatic drugs, *anti-TNF* anti-tumor necrosis factor

**Table 3:** Activity and functionality index average per visit

Clinical variable	Visit 1	Visit 2	Visit 3
DAS 28 CRP	3.23 (±1.39)	3.08 (±1.3)	3.03 (±1.23)
DAS 28 ESR	3.55 (±1.53)	3.37 (±1.42)	3.42 (±1.38)
CDAI	12.8 (±12.4)	10.3 (±10.5)	10.1 (±10.2)
HAQ	0.923 (±0.796)	0.83 (±0.739)	0.801 (±0.709)

*DAS 28 ESR, DAS 28 CRP* disease activity score 28 ESR and CRP, *CDAI* clinical disease activity index, *HAQ* health assessment questionnaire

Over the observation period, patients had 92 infection events and urinary tract was the most common site, followed by lower respiratory tract (Table 4). Regarding SI, 70 patients (8.32%) had 89 events, corresponding to 13 SI per 100 patient-years. Five of these patients had three SI events and nine had two. After six months, 45 (6.5%) patients presented with some SI, and in the last six months of follow-up, 30 (4.3%). No case of sepsis was documented.

**Table 4:** Infectious adverse event per visit

Variable	Visit 2 N (%) (n = 696)	Visit 3 N (%) (n = 691)
Severe infection since last visit	45 (6.5%)	30 (4.3%)
Infections since last visit	55 (7.9%)	37 (5.3%)
Upper airway infection	6 (0.9%)	5 (0.7%)
Lower airway infection	13 (1.9%)	7 (1%)
Urinary tract infection	17 (2.4%)	11 (1.6%)
Bone or joint infection	4 (0.6%)	4 (0.6%)
Skin Infection	9 (1.3%)	5 (0.7%)
Sepsis	0 (0%)	0 (0%)
Other infections	6 (0.9%)	5 (0.7%)

*N* number of observations, *%* percentage

After the univariate analysis (Table S1), we excluded from the subsequent analysis the presence of smoking or alcoholism, age, sex, gastrointestinal tract diseases, neoplasms, history of previous infection, obesity, and number of swollen joints for presenting *p* values ≥ 0.20. The final multivariate model (Table 5) showed that after six months a patient is 2.2 times (95% CI: 1.8–2.8) more likely to have a SI. The presence of pulmonary fibrosis, chronic kidney disease (CKD) and central nervous system (CNS) disease increased the chances of SI by 3.2 times (95% CI: 1.5–6.9), 3.6 times (95% CI: 1.2–10.4) and 2.4 times (95% CI: 1.2–5.0), respectively, with CNS disease including hemiplegia, Parkinson disease, dementia and psychiatric illness. The daily use of corticosteroids in doses equal to or higher than to 15 mg of prednisolone (or equivalent) increased the chances by 5.4 times (95% CI: 2.3–12.4), and for each increase of 1 unit in the HAQ, the chance of presenting SI increased by 60% (95% CI: 20–120%). The association between corticosteroid use and SI was dose-dependent until daily doses equal to or higher than 15 mg of prednisolone (or equivalent) in which the highest association was found.

**Table 5:** Multivariable analysis

Variable	Coef.	OR (95% CI)	*p*-value
Time (Visit)	0.81	2.2 (1.8–2.8)	<0.001
Pulmonary fibrosis	1.17	3.2 (1.5–6.9)	0.003
Chronic kidney disease	1.27	3.6 (1.2–10.4)	0.02
CNS diseases	0.89	2.4 (1.2–5.0)	0.015
Prednisolone (or equivalent) ≥ 15 mg/day	1.68	5.4 (2.3–12.4)	<0.001
HAQ	0.46	1.6 (1.2–2.2)	0.005

*Coef* coefficients, *OR* odds ratio, *95%CI* 95% confidence interval, *CNS* central nervous system diseases, *HAQ* health assessment questionnaire

Evaluating the groups of comorbidities, the presence of cardiovascular disease, pulmonary disease, metabolic disease, CKD and CNS disease were those that had a statistical association with SI in the initial univariate analysis. When performing further univariate analysis (Table S2), we observed that “other coronary diseases” presented a p value lower than 0.05, as well as chronic obstructive pulmonary disease (COPD), CKD classified as “moderate to severe” and the presence of “hemiplegia” and “psychiatric diseases”. Among the metabolic diseases, neither diabetes with nor without target organ damage alone had a relationship with the occurrence of infections.

## Discussion

As RA itself is known to be related to an increased risk of infection [24], it is of great interest to know which other factors presented by the patients may lead to an additional risk. Some of these factors can be specific to each population, with different socioeconomic conditions, management protocols and access to treatment and there is little data available about the South American population.

We found an incidence of 13 infections per 100 patient-years. In 2002, Doran [3] saw an incidence of 9.57 SI per 100 patient-years, whereas the incidence of all infection events in this cohort was 19.64 per 100 patient-years. More recently, in 2019, a large national US cohort [24] showed a high frequency (19.6 per 100 patient-years) of SI when following patients for long periods (15 years). However, in 2020, Wang observed 10.8 SI per 100 patients-year after a year of segment in an Australian cohort [25].

Our study associated the occurrence of SI events with the presence of comorbidities – such as pulmonary fibrosis, CKD, central nervous system diseases –, the use of moderate doses of corticosteroids, and reduced functionality as assessed by the HAQ. These results were similar to those already described in other cohorts [8–12, 26] and some of these factors can be associated, such as higher corticosteroid doses and pulmonary fibrosis. In a recent Taiwan cohort [27], Ng KH et al showed a relationship between corticosteroid daily use and mortality due to infection and disease activity in patients with pulmonary fibrosis associated with RA.

In contrast, factors that were also related to the occurrence of infectious events in other cohorts, such as disease duration, disease activity assessed by CDAI, elevation of inflammatory reactants and diabetes [11, 12, 24, 28] did not achieve statistical significance to be included in our final model. This can be justified by the homogeneity of the cohort sample, in which patients with moderate disease activity and with a long disease duration (14 years on average) predominated. Furthermore, the association between SI and diabetes was not always achieved in RA cohorts [15, 25, 29]. BIOBADABRASIL, Brazilian registry for biologic drugs, didn’t found association between diabetes and SI in patients with rheumatic disease either [15].

Regarding the use of corticosteroids, much effort has been done to identify a dose limit considered safe for use in the treatment of RA. Previous studies were able to show that the use of corticosteroids, even at low doses, such as equal to or less than 5 mg of prednisolone (or equivalent) per day, was associated with a higher risk of infections. This risk increases with the dose escalation of the medication [8, 11]. In this cohort, the association between corticosteroid use and SI also occurred in a dose-dependent manner, with the highest association found with doses equal to or higher than 15 mg of prednisolone (or equivalent). According to the most recent recommendations of the EULAR and ACR, if steroids are necessary as initial or bridging therapy, they should be discontinued as soon as possible [30, 31].

We found no statistically significant association between the use of any type of DMARD and the occurrence of SI. This finding differs from what is described in the literature [13–15, 29]. In the South American registry for biologic monitoring [32], Ranza et al. found an adjusted hazard ratio (HR) of 2.03 [1.05–30.9] comparing biologic DMARDs and synthetic DMARDs, and Quartuccio [29] observed that this risk was higher in the beginning of any biologic drug.

Reduced functionality assessed by the HAQ was another important factor associated with SI in the REAL cohort. Similarly, Weaver et al found a 30% increase in the risk of SI for every 0.4-unit HAQ-DI increase [9]. This metric can be influenced by pain, swollen joints, damage, deformities but also by fatigue and depression [33]. Previous studies showed that early treatment, tight disease control and biologic use can reduce progression of disability on RA [34, 35].

When performing the additional univariate analysis, “other coronary disease” and COPD were associated with SI with an OR of 7.48 (95% CI: 2.45–22.81) and 3.92 (95% CI: 1.78–8.59) respectively. The association between coronary disease and infections complications has already been described in literature, wherein certain infections agents have been implicated in atherosclerotic disease [36, 37]. Furthermore, SI is associated with 10-fold risk of 30-day death after an acute myocardial infarction [38] However, no causal relationship between chronic coronary disease and SI has been found and further studies are needed for better understanding this data. In turn, COPD is a known risk factor for SI and the risk for chronic infections and multidrug-resistant organisms is also higher compared to the general population [39].

The main limitation from this study is about selection bias, since data were collected only in tertiary centers. In these hospitals, there is a greater number of patients with severe and refractory diseases, for whom the use of biological medications and small molecules are available according to the national treatment protocol [39]. Theoretically, these patients with severe disease and a high level of immunosuppression form the group with the greater risk of SI [7, 9].

Furthermore, information was not available on some variables that could potentially influence access to the health system and the risk of SI such as color and education level. However, social level, another potential influencer of access to the healthcare system, was included and did not reach statistical significance (*p* > 0.05).

Given the important frequency of respiratory infections in this cohort, the lack of information on vaccination coverage is another limitation. Data was collected before the COVID19 pandemic, but airway infections such influenza, pneumococcus and Hemophilus B are important causes of SI that are potentially preventable with vaccination. In a systematic review, Furer et al showed a higher incidence and prevalence of vaccine preventable infection in patients with autoimmune inflammatory rheumatic disease (AIRD) compared to general population [40]. Other cohorts have shown a suboptimal vaccine coverage through patients with AIRD [41], even in tertiary centers [42, 43].

In addition, as this is an observational study, information and confounding biases are possible. This may justify the absence of sepsis diagnoses among SI and the small number of patients with a history of SI (16 patients) before the start of the follow-up. Another study limitation was the number of patients who did not attended all the visits (35%), as expected in a real-life study, in which losses are usually higher.

Despite those limitations, this study, as derived from a real-life nationwide cohort (REAL study), included a large representative population sample. Data assessing SI in this population in Brazil are still scarce.

## Conclusion

In conclusion, in the REAL cohort the administration of corticosteroids at moderate dosages increased the susceptibility to severe infections. Reduced functionality assessed by the HAQ and comorbidities were other important factors associated with SI in this cohort.

## Electronic supplementary material

Below is the link to the electronic supplementary material.

Supplementary Material 1: Univariate analysis

Supplementary Material 2: Additional univariate analysis

## Data Availability

The datasets used and/or analyzed during the current study are available from the corresponding author on reasonable request.

## Abbreviations

RA: Rheumatoid arthritis
SI: Serious infection
ACR: American college of rheumatology
EULAR: European league against rheumatism
EAMs: Extra-articular manifestations
DAS: Disease activity score
CDAI: Clinical disease activity index
HAQ: Health assessment questionnaire
anti-CCP: Anti-cyclic citrullinated peptide antibody
CRP: C-reactive protein
ESR: Erythrocyte sedimentation rate
AIDS: Acquired immunodeficiency syndrome
GEE: Generalized estimating equations
SD: Standard deviation
OR: Odds ratio
DMARDs: Disease-modifying antirheumatic drugs
CKD: Chronic kidney disease
CNS: Central nervous system
