# Supplementary material for: Predictors of serious infections in rheumatoid arthritis—a prospective Brazilian cohort
Source: Adv Rheumatol. 2024 Mar 29;64:23. doi: 10.1186/s42358-024-00363-1 (PMC13488804; doi:10.1186/s42358-024-00363-1)
Supplement: Supplementary file 1 [file 42358_2024_363_MOESM1_ESM.docx]

Supplementary 1 - Univariate analysis

| **Variable** | **Serious infection†** | |  | **Effect size^#^** |
| --- | --- | --- | --- | --- |
|  | **Yes**  **(n=75)** | **No**  **(n=2.153)** | **P-value** | **[95% IC]** |
| Visit |  |  | <0.001 | 2.1 [1.72 a 2.55] |
|  |  |  |  |  |
| Socioeconomic level, n (%) |  |  |  |  |
| A | 1 (1.4%) | 32 (1.5%) | 0.75 | 0.71 [0.09 a 5.80] |
| B1 | 4 (5.6%) | 91 (4.3%) | 0.96 | 0.97 [0.31 a 3.08] |
| B2 | 10 (13.9%) | 415 (19.6%) | 0.14 | 0.54 [0.24 a 1.24] |
| C1 | 17 (23.6%) | 616 (29.0%) | 0.19 | 0.62 [0.31 a 1.25] |
| C2 | 25 (34.7%) | 616 (29.0%) | 0.86 | 0.94 [0.48 a 1.83] |
| D-E | 15 (20.8%) | 353 (16.6%) |  | 1.00 |
| Missing information | 3 | 30 |  |  |
| Socioeconomic level (points), mean (SD) | 23±8.8 (21) | 24±7.9 (23) | 0.21 | 0.98 [0.94 a 1.02] |
| Smoking: ex-user or user, n (%) | 32 (42.7%) | 821 (38.1%) | 0.44 | 1.21 [0.76 a 1.94] |
| Smoking: no, n (%) | 43 (57.3%) | 1332 (61.9% |  | 1.00 |
| Alcoholism: ex-user or user, n (%) | 11 (14.7%) | 307 (14.3%) | 0.9 | 1.04 [0.55 a 1.99] |
| Alcoholism: no, n (%) | 64 (85.3%) | 1846 (85.7%) |  | 1.00 |
| Female gender, n (%) | 67 (89.3%) | 1934 (89.8%) | 0.941 | 1.03 [0.49 a 2.18] |
| Male gender, n (%) | 8 (10.7%) | 219 (10.2%) |  | 1.00 |
| Age (years), mean (SD) | 58±11.3 (58) | 56±11.4 (57) | 0.32 | 1.01 [0.99 a 1.03] |
| Age ≥ 60 years, n (%) | 31 (41.3%) | 857 (39.8%) | 0.84 | 1.05 [0.66 a 1.68] |
| Age < 60 years, n (%) | 44 (58.7%) | 1296 (60.2%) |  | 1.00 |
| Disease duration (years), mean (SD) | 17.9±11 (15.8) | 14.4±9.4 (12.4) | 0.003 | 1.03[1.01 a 1.05] |
| Presence of extra-articular manifestations: yes, n (%) | 23 (30.7%) | 356 (16.5%) | 0.002 | 2.23[1.34 a 3.70] |
| Presence of extra-articular manifestations: no, n (%) | 52 (69.3% | 1797 (83.5%) |  | 1.00 |
| Pulmonary fibrosis: yes, n (%) | 10 (13.3%) | 78 (3.6%) | <0.001 | 4.22 [2.08 a 8.55] |
| Pulmonary fibrosis: no, n (%) | 65 (86.7% | 2075 (96.4%) |  | 1.00 |
| Cardiovascular disease: yes, n (%) | 49 (65.3%) | 1116 (51.8%) | 0.016 | 1.82 [1.12 a 2.97] |
| Cardiovascular disease: no, n (%) | 26 (34.7%) | 1037 (48.2%) |  | 1.00 |
| Pulmonary disease: yes, n (%) | 13 (17.3%) | 174 (8.1%) | 0.006 | 2.41 [1.29 a 4.51] |
| Pulmonary disease: no, n (%) | 62 (82.7%) | 1979 (91.9%) |  | 1.00 |
| Gastrointestinal disease: yes, n (%) | 4 (5.3%) | 125 (5.8%) | 0.92 | 0.95 [0.34 a 2.64] |
| Gastrointestinal disease: no, n (%) | 71 (94.7%) | 2028 (94.2%) |  | 1.00 |
| Chronic kidney disease: yes, n (%) | 4 (5.3%) | 37 (1.7%) | 0.035 | 3.19 [1.09 a 9.37] |
| Chronic kidney disease: no, n (%) | 71 (94.7%) | 2116 (98.3%) |  | 1.00 |
| Metabolic diseases: yes, n (%) | 17 (22.7%) | 310 (14.4%) | 0.046 | 1.77 [1.00 a 3.12] |
| Metabolic diseases: no, n (%) | 58 (77.3%) | 1843 (85.6%) |  | 1.00 |
| Central nervous system diseases: yes, n (%) | 10 (13.3%) | 150 (7.0%) | 0.039 | 2.10 [1.03 a 4.24] |
| Central nervous system diseases: no, n (%) | 65 (86.7%) | 2003 (93.0%) |  | 1.00 |
| Neoplasm: yes, n (%) | 1 (1.3%) | 46 (2.1%) | 0.65 | 0.64 [0.09 a 4.62] |
| Neoplasm: no, n (%) | 74 (98.7%) | 2107 (97.9%) |  | 1.00 |
| History of previous infection: yes, n (%) | 1 (1.3%) | 34 (1.6%) | 0.96 | 0.95 [0.13 a 6.75] |
| History of previous infection: no, n (%) | 74 (98.7%) | 2119 (98.4%) |  | 1.00 |
| Obesity: yes, n (%) | 17 (22.7%) | 605 (28.6%) | 0.3 | 0.75 [0.43 a 1.30] |
| Obesity: no, n (%) | 58 (77.3%) | 1509 (71.4%) |  | 1.00 |
| Missing information | 0 | 39 |  |  |
| Seropositive disease: yes, n (%) | 66 (89.2%) | 1760 (82.7%) | 0.13 | 1.87 [0.84 a 4.14] |
| Seropositive disease: no, n (%) | 8 (10.8%) | 369 (17.3%) |  | 1.00 |
| Missing information | 1 | 24 |  |  |
| Age-adjusted Charlson Comorbidity Index, mean (SD) | 2.1±1.9 (2.0) | 1.78±1.5 (2.0) | 0.049 | 1.17 [1.00 a 1.37] |
| Charlson Comorbidity Index, mean (SD) | 0.77±1.4 (0) | 0.47±0.9 (0) | 0.004 | 1.32 [1.09 a 1.61] |
| Corticosteroid use: yes, n (%) | 38 (52.1%) | 1006 (47.4%) | 0.61 | 1.13 [0.70 a 1.80] |
| Corticosteroid use: no, n (%) | 35 (47.9%) | 1117 (52.6%) |  | 1.00 |
| Missing information | 2 | 30 |  |  |
| Corticosteroid dose* | 4.4±6.4 (2.5) | 2.8±3.7 (0.0) | 0.006 | 1.08 [1.02 a 1.14] |
| Corticosteroid dose (< 10 mg)*, n (%) | 61 (83.6%) | 1974 (93.0%) |  | 1.00 |
| Corticosteroid dose (≥ 10 mg)*, n (%) | 12 (16.4%) | 149 (7.0%) | 0.006 | 2.47 [1.29 a 4.71] |
| Corticosteroid dose (< 15 mg)*, n (%) | 64 (87.7%) | 2090 (98.5%) |  | 1.00 |
| Corticosteroid dose (≥ 15 mg)*, n (%) | 9 (12.3%) | 33 (1.5%) | <0.001 | 8.5 [3.80 a 18.80] |
| Synthetic DMARD: yes, n (%) | 64 (85.3%) | 1940 (90.7%) | 0.13 | 0.59 [0.31 a 1.16] |
| Synthetic DMARD: no, n (%) | 11 (14.7%) | 199 (9.3%) |  | 1.00 |
| Missing information | 0 | 14 |  |  |
| Exclusive synthetic DMARD: yes, n (%) | 35 (46.7%) | 1232 (57.2%) | 0.11 | 0.68 [0.43 a 1.8] |
| Exclusive synthetic DMARD: no, n (%) | 40 (53.3% | 921 (42.8%) |  | 1.00 |
| Biological DMARD and small molecules: yes, n (%) | 35 (51.5%) | 854 (40.8%) | 0.15 | 1.43 [0.90 a 2.30] |
| Biological DMARD and small molecules: no, n (%) | 33 (48.5%) | 1237 (59.2%) |  | 1.00 |
| Missing information | 7 | 62 |  |  |
| Anti-TNF: yes, n (%) | 18 (26.5%) | 448 (21.4%) | 0.41 | 1.26 [0.73 a 2.18] |
| Anti-TNF: no, n (%) | 50 (73.5%) | 1643 (78.6%) |  | 1.00 |
| Missing information | 7 | 62 |  |  |
| Exclusive biologicals and small molecules: yes, n (%) | 6 (8.0%) | 146 (6.8%) | 0.78 | 1.13 [0.48 a 2.66] |
| Exclusive biologicals and small molecules: no, n (%) | 69 (92.0%) | 2007 (93.2%) |  | 1.00 |
| Synthetic DMARD in combination with biologicals or small molecules: yes, n (%) | 29 (38.7%) | 708 (32.9%) | 0.38 | 1.24 [0.77 a 1.99] |
| Synthetic DMARD in combination with biologicals or small molecules: no, n (%) | 46 (61.3%) | 1445 (67.1%) |  | 1.00 |
| DAS 28 CRP (mg/dL), mean (SD) | 3.39±1.23 (3.21) | 3.12±1.32 (2.89) | 0.027 | 1.19 [1.01 a 1.39] |
| DAS 28 CRP - Remission, n (%) | 21 (32.3%) | 722 (41.5%) |  | 1.00 |
| DAS 28 CRP - Low activity, n (%) | 11 (16.9%) | 302 (17.4%) | 0.636 | 1.20 [0.57 a 2.52] |
| DAS 28 CRP - Moderate activity, n (%) | 25 (38.5%) | 541 (31.1%) | 0.105 | 1.65 [0.90 a 3.03] |
| DAS 28 CRP - High activity, n (%) | 8 (12.3%) | 174 (10.0%) | 0.177 | 1.79 [0.77 a 4.15] |
| Missing information | 10 | 414 |  |  |
| DAS 28 ESR (mm), mean (SD) | 3.94±1.29 (4.06) | 3.45±1.46 (3.35) | 0.009 | 1.40 [1.09 a 1.81] |
| DAS 28 ESR - Remission, n (%) | 6 (11.8%) | 482 (29.9%) |  | 1.00 |
| DAS 28 ESR - Low activity, n (%) | 10 (19.6%) | 243 (15.1%) | 0.027 | 3.22 [1.16 a 8.93] |
| DAS 28 ESR - Moderate activity, n (%) | 28 (54.9%) | 671 (41.7%) | 0.009 | 3.29 [1.33 a 8.10] |
| DAS 28 ESR - High activity, n (%) | 7 (13.7%) | 214 (13.3%) | 0.058 | 2.92 [0.95 a 8.91] |
| Missing information | 24 | 543 |  |  |
| CDAI, mean (SD) | 13.27±11.83(10.00) | 11.13±11.25 (8.00) | 0.02 | 1.03 [1.01 a 1.05] |
| CDAI - Remission, n (%) | 12 (16.0%) | 529 (24.6%) |  | 1.00 |
| CDAI - Low activity, n (%) | 29 (38.7%) | 755 (35.2%) | 0.145 | 1.67 [0.84 a 3.31] |
| CDAI - Moderate activity, n (%) | 21 (28.0%) | 556 (25.9%) | 0.136 | 1.73 [0.84 a 3.58] |
| CDAI - High activity, n (%) | 13 (17.3%) | 308 (14.3%) | 0.064 | 2.14 [0.96 a 4.78] |
| Missing information | 0 | 5 |  |  |
| CRP level (mg/dL), mean (SD) | 1.77±2.98 (0.50) | 1.39±3.08 (0.59) | 0.022 | 1.05 [1.01 a 1.09] |
| ESR level (mm), mean (SD) | 37.69±32.9 (29.00) | 28.92±24.94 (23.00) | 0.036 | 1.01 [0.99 a 1.03] |
| HAQ, mean (SD) | 1.16±0.83 (1.00) | 0.85±0.74 (0.75) | <0.001 | 1.80 [1.32 a 2.47] |
| Number of swollen joints in 28, mean (SD) | 2.08±4.1 (1.0) | 1.94±3.4 (0.0) | 0.53 | 1.02 [0.94 a 1.10] |
| Number of swollen joints in 46, mean (SD) | 2.12±4.1 (1.0) | 2.25±4.0 (0.0) | 0.95 | 1.00 [0.94 a 1.06] |
| Number of tender joints in 28, mean (SD) | 4.33±6.7 (2.0) | 3.40±5.9 (1.0) | 0.056 | 1.03 [0.99 a 1.07] |
| Number of tender joints in 46, mean (SD) | 5.04±8.4 (2.0) | 4.39±7.7 (1.0) | 0.2 | 1.02 [1.00 a 1.04] |

Legend: †Considering the sum of all valid visits (2.228) # Effect sizes: odds ratios and mean differences, for categorical and continuous variables respectively. * Prednisolone equivalent dose. N - number of observations. % - percentage. SD - standard deviation. DMARD - disease-modifying antirheumatic drugs; anti-TNF - anti-tumor necrosis factor; DAS 28 ESR, DAS 28 CRP - Disease Activity Score 28 ESR and CRP. CDAI - Clinical Disease Activity Index. CRP - C-reactive protein; ESR - Erythrocyte sedimentation rate; HAQ -Health Assessment Questionnaire.
