# Supplementary material for: Predictors of serious infections in rheumatoid arthritis—a prospective Brazilian cohort
Source: Adv Rheumatol. 2024 Mar 29;64:23. doi: 10.1186/s42358-024-00363-1 (PMC13488804; doi:10.1186/s42358-024-00363-1)
Supplement: Supplementary file 2 [file 42358_2024_363_MOESM2_ESM.docx]

Supplementary 2 - Additional univariate analysis

| **Comorbidities** | **Coef.** | **P- value** | **OR** | **95% IC** |
| --- | --- | --- | --- | --- |
|  |  |  |  |  |
| Cardiovascular disease | 0.6 | 0.016 | 1.82 | 1.12 a 2.97 |
| Cerebrovascular disease | -0.67 | 0.51 | 0.51 | 0.07 a 3.82 |
| Peripheral vascular disease | -38.28 | 0.999 | 0 | 0.00 a 0.00 |
| Myocardial infarction | 0.4 | 0.59 | 1.49 | 0.35 a 6.40 |
| Systemic arterial hypertension | 0.47 | 0.053 | 1.6 | 0.99 a 2.57 |
| Congestive heart failure | 0.21 | 0.78 | 1.23 | 0.29 a 5.21 |
| Other heart disease | 0.94 | 0.084 | 2.55 | 0.88 a 7.40 |
| Other coronary disease | 2.01 | <0.001 | 7.48 | 2.45 a 22.81 |
| Lung disease | 0.88 | 0.006 | 2.41 | 1.29 a 4.51 |
| Asthma | -0.25 | 0.73 | 0.78 | 0.19 a 3.25 |
| COPD | 1.37 | <0.001 | 3.92 | 1.78 a 8.59 |
| Other lung disease | 0.98 | 0.072 | 2.66 | 0.91 a 7.74 |
| Chronic Kidney disease | 1.16 | 0.035 | 3.19 | 1.09 a 9.37 |
| Mild | 0.52 | 0.61 | 1.68 | 0.23 a 12.38 |
| Moderate or severe | 1.48 | 0.024 | 4.38 | 1.21 a 15.77 |
| Metabolic disease | 0.57 | 0.046 | 1.77 | 1.00 a 3.12 |
| Diabetes with target organ damage | 0.7 | 0.26 | 2.01 | 0.60 a 6.74 |
| Diabetes without target organ damage | 0.5 | 0.1 | 1.65 | 0.91 a 3.02 |
| CNS disease | 0.74 | 0.039 | 2.1 | 1.03 a 4.24 |
| Hemiplegia | 2.74 | 0.051 | 15.47 | 0.99 a 242.94 |
| Dementia | -36.22 | 0.999 | 0 | 0.00 a 0.00 |
| Parkinson | - | - | - | - |
| Psychiatric illness | 0.76 | 0.033 | 2.14 | 1.06 a 4.31 |

Legend: COPD - chronic obstructive pulmonary disease; CNS - central nervous system
